# Effect of an Eclectic Physiotherapeutic Approach on Physical Performance, Fatigue, and Hospital Anxiety and Depression in a Patient Receiving Radiotherapy for Squamous Cell Carcinoma of the Buccal Mucosa: A Case Report

**DOI:** 10.7759/cureus.86172

**Published:** 2025-06-16

**Authors:** Tushara Nair, G. Palani Kumar

**Affiliations:** 1 College of Physiotherapy, Sumandeep Vidyapeeth (Deemed to be University), Vadodara, IND

**Keywords:** carcinoma buccal mucosa, physiotherapy intervention, recreational therapy, squamous cell carcinoma (scc), yoga therapy

## Abstract

Squamous cell carcinoma of the buccal mucosa is one of the most common tumors among head and neck cancers. It first involves the superficial layer of the buccal mucosa and then slowly starts infiltrating into the deeper tissues as well. A 45-year-old female patient diagnosed with squamous cell carcinoma of the right buccal mucosa underwent a marginal mandibulectomy with pectoralis major myocutaneous flap reconstruction and was admitted for radiotherapy sessions. She also had a history of fatigue and decreased physical performance. A personalized physiotherapy protocol was formulated, which included general mobility exercises, strengthening exercises, light aerobics, yoga, relaxation, and recreational activities. The Timed Up and Go Test was used to assess physical performance, the Functional Assessment of Chronic Illness Therapy Fatigue Scale was used to assess cancer-related fatigue, and the Hospital Anxiety and Depression Scale was used to assess the anxiety and depression due to hospital admission. This case study concluded that this personalized eclectic approach definitely improves physical performance, decreases fatigue, anxiety, and depression.

## Introduction

Squamous cell carcinoma of the buccal mucosa is a tumor of the oral cavity where the topmost layer of the mucosa, which consists of thin and flat cells, divides uncontrollably and starts infiltrating the deeper tissues [1,2]. Epidemiologic studies reveal that head and neck cancers are the sixth most common type globally, whereas the third most common in South Central Asia [3,4]. Oral cancer accounts for 30% of all cancer cases in India due to an increase in addiction to cigarettes and tobacco. The prevalence of oral squamous cell carcinoma with oral submucous fibrosis has been reported to be around 13.7% [5-7].

Patients with buccal mucosa tumors generally present with ulcers with raised, white or red patches in the oral cavity, which are painful and can bleed. Patients may have swelling in the region of the jaw, neck, loose teeth, halitosis, and difficulty opening the mouth [2,6]. The main line of treatment is surgery, with additional therapies including chemotherapy and radiation therapy [8]. All these available treatment options lead to a decrease in the functional capacity and physical performance of individuals [9]. Cancer-related fatigue (CRF), depression, and anxiety are also common problems after active cancer treatment [10]. As these patients are admitted for a long time in the wards for treatment, it adds to cancer-related depression and anxiety [11].

Physiotherapy plays an integral role in improving physical performance and decreasing depression and anxiety in such patients. Physiotherapy generally includes mobility exercises, strengthening exercises, light aerobics, physio-yoga, and recreational activities [12,13]. Many studies have proven the individual effects of aerobics, strengthening, or yoga, but there is a lack of an eclectic approach involving multiple aspects of physiotherapy.

Hence, this study aims to see whether an eclectic physiotherapeutic approach, including mobility and strengthening exercises, light aerobics, physio-yoga, and recreational activities, helps improve the physical performance and hospital-based anxiety and depression in a patient with buccal mucosa tumor undergoing radiotherapy.

## Case presentation

Patient information

A 45-year-old female, a home-maker, residing in a small village, had a history of a 5 cm swelling inside the mouth in the right cheek region for nine months, which slowly increased in size. With these complaints, she had visited a tertiary care hospital and was diagnosed with a stage IV right buccal mucosa tumor. She had undergone three cycles of a chemotherapy regimen, including cisplatin and fluorouracil. Subsequently, she was operated in the form of tumor excision and a marginal mandibulectomy, with pectoralis major myocutaneous flap reconstruction four months after chemotherapy. After the surgery, she was advised to undergo radiotherapy as the tumor was classified as T3-T4, for which she had visited Dhiraj General Hospital (DGH). One and a half months after the surgery, the patient was admitted to the radiotherapy ward of DGH, where she received radiotherapy, 60 Gy in 2 Gy fractions for five days a week for seven weeks. The inpatient physiotherapy sessions commenced during this duration and continued for one month, following which she was discharged. At the time of discharge, the patient was fully functional and independent in her daily activities.

Clinical findings

Informed consent was taken from the patient, after which a detailed physiotherapy assessment was conducted. The physiotherapy assessment revealed that the patient had a positive history of tobacco chewing for 10 years. The observational findings suggested that the patient had a slouched posture, with forward head, rounded shoulders, and kyphosis. The patient also had a scar at the surgical site, which was mobile. The neck and shoulder range on the right side was terminally restricted. The ranges of other joints were full and free. The muscle strength of all the muscles of the shoulder and neck was good within the available range. The Physical Activity Readiness Questionnaire (PAR-Q) was filled out before commencing the session. The patient answered No to all the questions. The Timed Up and To Test (TUG) was used to assess physical performance, the Functional Assessment of Chronic Illness Therapy Fatigue (FACIT-F) Scale was used to assess CRF, and the Hospital Anxiety and Depression Scale (HADS) was used to assess anxiety and depression due to hospital administration. The timeline of events is presented in Table 1.

**Table 1:** Timeline of events.

Events	Date of events
History of swelling	Since 9 months
Diagnosis of a right buccal mucosa tumor	On the first visit to the hospital.
Date of chemotherapy commencement	10 days after the diagnosis of tumor
Date of surgery	Four months after chemotherapy
Date of admission to the hospital for radiotherapy	One and a half months after the surgery
Date of radiotherapy commencement	After the second day of admission to the hospital
Date of physiotherapy commencement	21 days after the patient was admitted for radiotherapy
Date of discharge	One month after the commencement of physiotherapy

Eclectic physiotherapy approach

An eclectic physiotherapeutic approach was used in the treatment, wherein a personalized physiotherapy protocol was formulated for the patient, which included physiotherapy exercises in the form of general mobility and strengthening, light aerobics, relaxation exercises, yoga and meditation, fun activities, etc. A total of 20 sessions were conducted, five sessions of 30 minutes per week for four weeks. The protocol of the physiotherapy session is illustrated in Table 2.

**Table 2:** Eclectic physiotherapeutic approach.

Day	Duration	Protocol
Monday	30 minutes	General mobility of all joints, which included the active movements available at all the joints, closed-chain strengthening exercises for the lower limb and upper limb, squats, lunges, and wall push ups (3 sets of 10 repetitions each)
Tuesday	30 minutes	Yoga included sukshmakriyas (active movements of all joints along with breathing), asanas (dandasana, jaanushirasana, setubandhasana, ek pada uttanpadasana, uttanasana, tadasana, etc.) (3 times with a 10-second hold) and pranayama (anulom-vilom and shivanand shwasan) (5 cycles each)
Wednesday	30 minutes	Light aerobics which included 10 minutes of warm-up exercises (general body movements and stretching), 10 minutes of light step aerobics, and 10 minutes of cool-down exercises (general body movements and stretching) (3 sets of 10 repetitions each)
Thursday	30 minutes	Relaxation exercises included Jacobson’s relaxation technique and Laura Mitchell technique (3 sets with a 10-second hold), Aumkar meditation (10 minutes)
Friday	30 minutes	Recreational activities like passing a ball, throwing and catching a ball, musical chair, etc. (performed in a group)

Outcome measures and results

**Figure 1:**
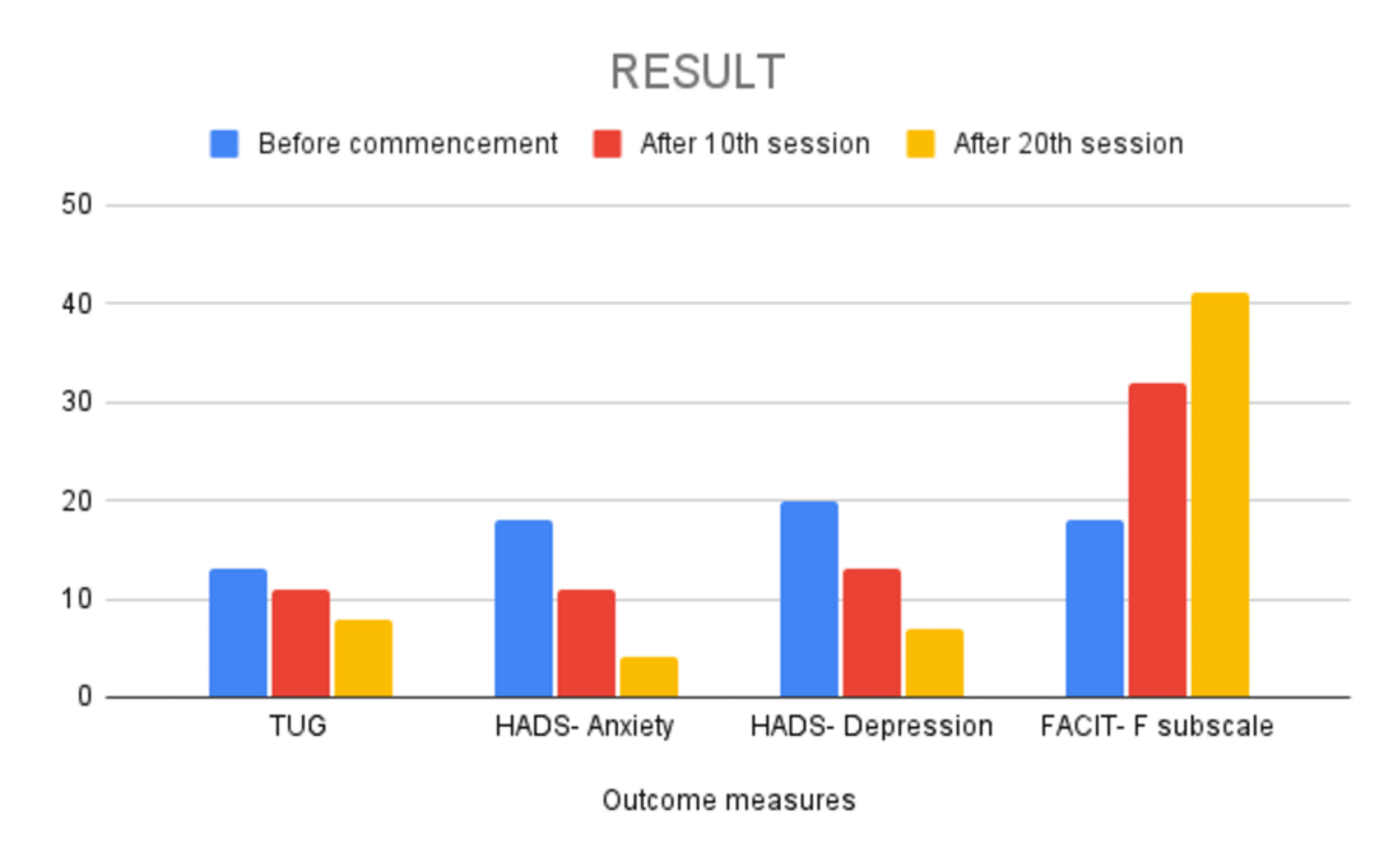
Bar diagram indicating improvements in the outcome measures. TUG: Timed Up and Go Test; HADS: Hospital Anxiety and Depression Scale; FACIT-F: Functional Assessment of Chronic Illness Therapy-Fatigue Scale

The physical performance was assessed using TUG, HADS was used to assess the depression and anxiety due to hospital admission, and FACIT-F was used to assess fatigue. The outcome measures were assessed before the commencement of treatment, after 10 sessions, and at the end of 20 sessions (Table 3, Figure 1).

**Table 3:** Results of the protocol on the outcome measures. TUG: Timed Up and Go Test; HADS: Hospital Anxiety and Depression Scale; FACIT-F: Functional Assessment of Chronic Illness Therapy-Fatigue Scale

Outcome measures	Before commencement	After the 10th session	After the 20th session
TUG	13	11	8
HADS-Anxiety	18	11	4
HADS-Depression	20	13	7
FACIT-F subscale	18	32	41

The above results conclude that the TUG, HADS-Anxiety, and HADS-Depression scores decreased after the 10th and 20th sessions, indicating improvement. Additionally, the FACIT-F score increased after the 10th and 20th sessions, also indicating improvement.

## Discussion

This case report discusses the effect of an eclectic physiotherapeutic approach on physical performance, fatigue, and hospital-based anxiety and depression in a 45-year-old female patient with a buccal mucosa tumor undergoing palliative care treatment in the form of radiotherapy.

Tobacco chewing is one of the common risk factors for buccal mucosa tumors. This patient also had a history of tobacco chewing for 10 years. A review by Ford and Rich on tobacco use and oral health concluded that if the oral mucosa is exposed to tobacco or its products, there are higher chances of developing benign, potentially malignant, and malignant tumors [14].

In this patient, a marginal mandibulectomy along with pectoralis major myocutaneous flap reconstruction was performed, after which radiation therapy was continued. Pradhan conducted a review of surgery for cancer of the buccal mucosa and concluded that marginal mandibulectomy is one of the frequently performed surgeries for buccal mucosa tumors. In addition, postoperative radiation therapy is also used [15].

Physiotherapy aims to increase the physical activity level, which helps improve physical activity and reduce fatigue, depression, and anxiety [16]. Cheville et al. assessed the effects of a structured physiotherapy rehabilitation program for cancer patients undergoing adjuvant therapy, and positive effects were noted on physical well-being [17]. This case study also included a multimodal structured rehabilitation program, and positive effects were found on physical performance, fatigue, and hospital-based depression and anxiety.

Light aerobics and resistance exercises help improve the physical fitness of cancer survivors. Christina et al. studied the effects of aerobic and resistance exercises on physical fitness in breast cancer survivors and concluded that there was a significant improvement in physical fitness and quality of life. Similarly, in this case study, light aerobics and strengthening exercises were performed, which led to a significant improvement in the quality of life [18].

Yoga, which is based on the mind-body concept, helps reduce anxiety and depression by maintaining a balance between the sympathetic and parasympathetic nervous systems. Liu et al. investigated patients receiving adjuvant chemotherapy for breast cancer, where the participants received yoga therapy. They concluded that mindfulness yoga helps decrease anxiety and depression [13].

Relaxation is an important factor to reduce CRF, and progressive muscle relaxation, which is a technique of exercise therapy, is very helpful in inducing relaxation was used in this case study. Wang et al. also concluded in a meta-analysis that progressive muscle relaxation helps improve CRF, anxiety, depression, and quality of life in cancer patients [19].

Hence, a multimodal treatment protocol was formulated for this patient, which included general mobility, strengthening, aerobics, yoga, relaxation, and recreational activities, which was found to be effective in improving the physical performance and reducing CRF, anxiety, and depression in this patient with a buccal mucosa tumor.

## Conclusions

Physiotherapy has been found to be effective in patients with cancer. In this case study, different aspects of physiotherapy were included, and an eclectic approach was formulated, which included general mobility, strengthening, light aerobics, yoga, relaxation, and recreational activities. This multimodal treatment program was effective in improving the physical performance and reducing fatigue, anxiety, and depression in this patient receiving radiotherapy after a marginal mandibulectomy in a known case of squamous cell carcinoma of the buccal mucosa.
